# Targeting Metastasis with Snake Toxins: Molecular Mechanisms

**DOI:** 10.3390/toxins9120390

**Published:** 2017-11-30

**Authors:** Félix A. Urra, Ramiro Araya-Maturana

**Affiliations:** 1 Anatomy and Developmental Biology Program, Institute of Biomedical Sciences, University of Chile, Independencia 1027, Casilla 7, Santiago 7800003, Chile; 2 Geroscience Center for Brain Health and Metabolism, Independencia 1027, Casilla 7, Santiago 7800003, Chile; 3 Instituto de Química de Recursos Naturales, Universidad de Talca, Casilla 747, Talca 3460000, Chile

**Keywords:** anti-cancer agents, cancer cells, invasion, migrastatic drugs, snake venom

## Abstract

Metastasis involves the migration of cancer cells from a primary tumor to invade and establish secondary tumors in distant organs, and it is the main cause for cancer-related deaths. Currently, the conventional cytostatic drugs target the proliferation of malignant cells, being ineffective in metastatic disease. This highlights the need to find new anti-metastatic drugs. Toxins isolated from snake venoms are a natural source of potentially useful molecular scaffolds to obtain agents with anti-migratory and anti-invasive effects in cancer cells. While there is greater evidence concerning the mechanisms of cell death induction of several snake toxin classes on cancer cells; only a reduced number of toxin classes have been reported (i.e., disintegrins/disintegrin-like proteins, C-type lectin-like proteins, C-type lectins, serinproteases, cardiotoxins, snake venom cystatins) as inhibitors of adhesion, migration, and invasion of cancer cells. Here, we discuss the anti-metastatic mechanisms of snake toxins, distinguishing three targets, which involve (1) inhibition of extracellular matrix components-dependent adhesion and migration, (2) inhibition of epithelial-mesenchymal transition, and (3) inhibition of migration by alterations in the actin/cytoskeleton network.

## 1. Introduction

Currently, anticancer therapies target the uncontrolled clonal proliferation of cancer cells with cytostatic drugs, which are an effective therapeutic strategy for certain cancer types such as hematological malignancies. However, in solid cancers, the proliferation is accompanied by the ability to invade and execute metastasis, involving different molecular mechanisms that are not inhibited or affected by conventional anti-cancer drugs. Therefore, to search for and design specific drugs to inhibit invasion and metastasis for treatment of solid cancers is a highly relevant issue [1].

The composition of solid tumors is heterogeneous, having several cancer cell subpopulations with different tumorigenic properties [2]. In a tumor, cancer cells acquire mutations that confer them with different proliferative capacities and survival advantages. A subpopulation, named metastasis-initiating cells (MICs), exhibits high plasticity to adapt their metabolic and proliferative requirements, ability to enter and exit dormancy state, and resistance to apoptosis and immune evasion, which is responsible for metastatic growth [3]. For example, during the initial steps of tumor growth of cancer cells confined to epithelium, certain colonies of malignant cells can form a carcinoma in situ separated from the stroma. In some cells, mutations provide the ability to establish a physical relationship with stroma and changes in extracellular signals from the microenvironment, triggering the secretion of soluble factors by stromal and hematopoietic cells [4,5] and inducing phenotypic changes in cancer cells known as epithelial–mesenchymal transition (EMT). This process recapitulates properties displayed by tissues during the embrionary development [6] that facilitate the dissociation of cancer cell from the tumor bulk and dissemination to distant organs, being considered a prerequisite for invasion and metastasis [2,7].

Metastasis is a complex process in which cancer cells disseminate from a primary tumor to invade a distant organ, this ability characterizes the tumor malignancy [6]. It has been described that about 90% of cancer-related deaths are caused by a metastatic disease [8]. It is clear that dissemination to specific organs depends upon blood flow patterns and of the relationship of the migrating cells with distant organ microenvironments, the stromal cell content, vascular architecture, presence of growth factors, metabolic substrates, and signaling molecules. These characteristics can be permissive or antagonistic to metastatic colonization, determining whether these cells grow to form secondary tumors [9].

The detailed mechanistic insight of the metastatic process contrast with the minimal progress in the identification of effective therapeutic targets and in the design of new anti-metastatic drugs [1]. Based on structural characteristics and their known interactions with macromolecules, toxins isolated from snake venoms may represent a natural source of molecular scaffolds to obtain agents with anti-migratory and anti-invasive effects in cancer cells. In this review, we summarize recent evidence on the inhibitory effect of snake toxins on adhesion, migration, and invasion of cancer cells.

## 2. Snake Toxins as Inhibitors of Cancer Metastasis

**Figure 1:**
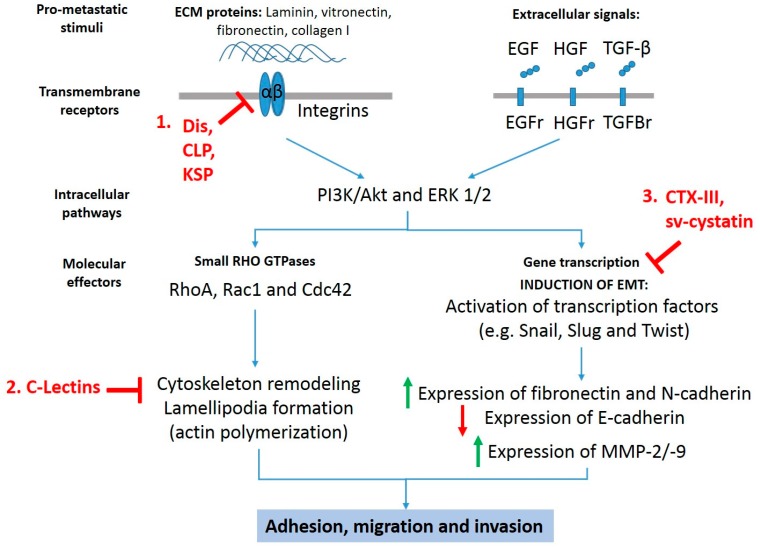
**Anti-metastatic targets for snake toxins.** Snake toxins inhibit pro-migratory and pro-invasive signals stimulated by extracellular matrix proteins and growth factors such as epidermal growth factor (EGF), hepatocyte growth factor (HGF), and transforming growth factor beta (TGF-β) though (1) inhibition of extracellular matrix (ECM) components-dependent adhesion and migration, (2) inhibition of migration by alterations in the actin/cytoskeleton network, and (3) inhibition of epithelial–mesenchymal transition (EMT). Dis: disintegrins; CLP: C-type lectin-like proteins; KSP: Kunitz-type serinprotease; C-Lectins: C-type lectins; CTX-III: cardiotoxin III; Sv-cystatin: snake venom cystatin.

There is ample literature showing that several isolated or recombinant snake venom toxins exhibit anti-cancer effects in vitro and in vivo preclinical models, inducing cell death via mitochondrial apoptotic pathway (intrinsic pathway) or necrosis [10,11,12]. In addition, certain toxins such as snake venom metalloproteases (SVMPs), disintegrins, phospholipases A2, C-type lectins (CLP), vascular apoptosis inducing proteins, and L-amino acid oxidases are able to inhibit angiogenesis [13,14,15] and activate the immune response during tumorigenesis [16]. While greater evidence on mechanisms of death induction of snake toxins on cancer cells have been reported, reduced information on the inhibitory mechanisms of adhesion, migration, and invasion of metastatic cancer cells is available. Despite the aforementioned information, it is possible distinguish three anti-metastatic mechanisms exhibited by at least six different snake toxin classes (Figure 1): involving (1) inhibition of extracellular matrix components (ECM)-dependent adhesion and migration, (2) inhibition of epithelial-mesenchymal transition, and (3) inhibition of migration by alterations in the actin/cytoskeleton network.

## 3. Inhibition of Extracellular Matrix Component-Dependent Adhesion and Migration

During the initial steps of metastasis, it is required the interaction between ECM components and cancer cell, involving the ability of these cells to adhere to ECM components and migrate through them [17]. Integrins are the major receptor family present on the cell surface for adhesion to the ECM and include heterodimeric, transmembrane glycoproteins composed of α and β subunits [18], whose dimerization leads to 24 integrin pairs with distinct extracellular ligand-binding specificities [18]—such as collagen, laminin, vitronectin, and fibronectin—through the tripeptide motif Arg-Gly-Asp = RGD [19]. Abundant evidence has correlated the increased overexpression of certain integrins αvβ3, α5β1, and αvβ6 with cancer progression [20,21,22]. Integrins activate intracellular signaling that control cytoskeleton organization, cell polarity, and formation of leading edge of migrating cancer cells [22], being an attractive anti-cancer target for new antagonist molecules [23,24].

Three toxin classes (snake venom disintegrins, C-type lectin-like protein, and Kunit-like serinprotease inhibitor) have been reported with anti-migratory effect mediated by interaction with integrins in cancer cells, which are summarized in Table 1.

Snake venom disintegrins are small non-enzymatic proteins mostly derived from proteolytic processing of precursors that contain a metalloprotease domain, known as snake venom metalloproteases (SVMPs), which are phylogenetically related with ADAMs (a disintegrin and metalloprotease) [25,26,27,28]. This protein family, commonly found in the venoms of the Viperidae snakes [26] and some rear-fanged snakes [28,29,30,31,32,33,34,35], is classified according to their modular architecture with multiple non-catalytic domains in SVMP P-I, P-II, and P-III classes. Disintegrins are derived from proteolytic processing of P-II SMVP class and usually exhibit the canonical “RDG” integrin-recognition motif; however, non-canonical integrin-binding motif—such as “MLD”, “KTS”, and “VGD”—are exhibited in some snake venom disintegrins [36,37]. In addition, proteolysis from P-III SVMP class originates disintegrin-like proteins, which have covalently bound the “disintegrin-like” and “cysteine (Cys)-rich” domains [27]. Comprehensive classification and structural characteristics of SVMP are found in Takeda et al., 2012 [27] and Takeda, 2016 [38].

A disintegrin isolated from the venom of the Middle American rattlesnake (*Crotalus simus tzabcan*) named tzabcanin [39], which has 71 amino acids and contains the canonical RGD-binding domain, exhibits a weak or null cytotoxic effect on cancer cell lines [39], but remarkable inhibitory effect of fibronectin- and vitronectin-dependent cell adhesion. This toxin binds αvβ3-integrins, which is the main receptor of the ECM protein vitronectin, inhibiting the adhesion and migration of melanoma and lung cancer cells [40].

DisBa-01, a recombinant RGD-disintegrin produced from a cDNA venom gland library of *Bothrops alternatus*, inhibits in vivo angiogenesis and pulmonary metastasis [49]. In oral squamous carcinoma cells, DisBa-01 selectively decreases the migration speed and directionality of fibronectin-stimulated migration, increasing the adhesion area and rate of adhesion maturation. It lacks effects on migration of non-malignant cells such as fibroblasts. DisBa-01 exhibits a high affinity on fibronectin binding receptor αvβ3 integrin [43]. Other recombinant disintegrins from Viperidae species have been reported such as αvβ3 integrin antagonists, inhibiting the migration of cancer cells (Table 1). Additional disintegrins and disintegrin-like proteins from snake venoms reported with anti-cancer effect can be found in Selistre-de-Araujo et al., 2010 [50].

Interestingly, Lebecin, and PIVL isolated from *Macrovipera lebetina* venom, which belong two different toxin classes C-type lectin-like protein and Kunitz-type serin protease inhibitor, respectively, exhibit inhibitory effect on fibrinogen- and fibronectin-stimulated adhesion and migration.

Lebecin is a C-type lectin-like protein with α and β subunits of 129 and 131 amino acids, respectively [47]. In triple-negative breast cancer MDA-MB-231 cells, lebecin does not affect the viability. However, it inhibits the fibrinogen- and fibronectin-dependent adhesion and migration in a dose-dependent manner [47]. It has been described that lebecin interacts with αvβ3 integrin; but based on the high identity of its amino acid sequence with other C-type lectin-like protein previously reported from *Macrovipera lebetina* venom with inhibitory effect on adhesion, migration, and invasion of cancer cells [51,52], it has been suggested that lebecin can block other integrins such as α5β1 [47].

PIVL is a monomeric polypeptide chain bound by three disulfide linkages, which inhibits trypsin activity and lacks effects on the viability but blocks αvβ3 integrin-dependent migration, affecting the motility and cell directionality persistence of cancer cells [48]. PIVL also exhibits in vitro and in vivo anti-angiogenic effects [53].

## 4. Inhibition of Epithelial–Mesenchymal Transition

Epithelial–mesenchymal transition (EMT) is a process in which epithelial cells transdifferentiate into mesenchymal cells, losing their morphoinmunophenotypic characteristics. Interestingly, EMT occurs in normal and healthy tissues during angiogenesis and lymphangiogenesis; but in certain pathological conditions such as chronic inflammation, fibrosis and cancer is reactivated [6]. In tumors, EMT-like transitions involve the loss of components related with cell-cell interactions, apico-basal cell polarity and reorganization of cytoskeleton. Cancer cells with EMT have tumorigenic properties that non-EMT cells do not exhibit, such as a high migratory state that promote invasion and metastasis [4,5], lacking response to signals of oncogene-induced senescence [54] and resistance to anti-cancer drugs [55,56,57].

EMT can be induced by growth factors such as transforming growth factor beta (TGF-β), epidermal growth factor (EGF), hepatocyte growth factor (HGF), insulin-like growth factors 1 and 2 [40], activating RAS, Notch, and Wnt signalings which have been associated with poor prognosis and cancer progression [58,59]. During EMT, there is a reduction of the epithelial marker E-cadherin and an increase of the expression of mesenchymal markers vimentin, N-cadherin [60], as well as activation of transcription factors Snail, Slug, Twist, which act as repressor of E-cadherin [5,61].

Cardiotoxin III (CTX-III), a membrane toxin from Taiwan cobra (*Naja naja*) venom [62], inhibits the migration of cancer cells by reversion of EGF- and HGF-induced EMT. Previously, CTX-III has been described as a potent inductor of cell death in several human cancer cell lines [63,64,65] and a migration inhibitor of oral and breast cancer cells through activation of JNK and p38, without effect on ERK signaling, producing decreased metalloproteases-2 and -9 (MMP-2/-9) levels [66,67].

In breast cancer cells, the paracrine role of epidermal growth factor (EGF) and its receptor EGFR (ErbB-1) contribute to invasion, intravasation, and metastasis [68] through activation of extracellular signal-regulated kinase 1/2 (ERK1/2), STAT3, or PI3K/Akt signaling, promoting the EMT [69,70,71]. CTX-III inhibits the EGF-induced EMT in breast cancer cells, reducing EGFR phosphorylation and activation PI3K/Akt and ERK1/2. It reduces the MMP-9 levels [72] and the mesenchymal markers vimentin and N-cadherin and increases E-cadherin levels, inhibiting EGF-induced invasion and migration [72,73]. A similar effect of CTX-III on hepatocyte growth factor (HGF)-stimulated migration and invasion in breast cancer cells has been described [73,74,75].

Cancer cells can excrete cysteine-cathepsins, which are endopeptidases located intracellularly in endolysosomal vesicles [76] that are essential during the breakdown the ECM to promote the invasion and metastasis [77]. During EMT, cancer cells exhibit an increased extra- and intra-cellular proteolysis mediated by cathepsins, matrix metalloproteinases, urokinase-type plasminogen activator (uPA), and serinproteases such as kallikreins [78]. This proteolytic activity removes surface molecules involved in cell adhesion such as E-cadherin [79,80], limiting the cell–cell interaction and remodeling the extracellular matrix to uncover binding epitopes recognized by integrins and to form trials for cell migration [81]. Cysteine-cathepsins are regulated by natural inhibitors such as cystatins [82], which represent a group constituted by three types (type 1-stenfins, type-2 cystatins, type 3-kininogens) of cystatin domain containing proteins [83]. From *Naja naja atra* venom, it has been isolated a snake venom cystatin (Sv-cystatin) that exhibits a shorter sequence than other type-2 cystatins, such as cystatin M and cystatin C [84]. For this snake toxin, inhibitory effects on invasion and metastasis mediated by reduction of EMT markers has been described in MHCC97H liver cancer cells [85]. Sv-cystatin decreases the cathepsin B activity, MMP-2, and MMP-9 levels, increasing E-cadherin and decreasing EMT proteins N-cadherin and twist [85].

## 5. Alterations in the Actin/Cytoskeleton Network

During migration and invasion of cancer cells, the actin cytoskeleton is remodeled under extracellular stimuli, which is mediated by several receptors, including integrins [19]. Small GTPases Rho, Rac, and Cdc42 participate in the intracellular signaling involved in the control of the actin cytoskeleton architecture required for cell motility in individual and collective migration [86], which is a common signaling for normal and cancer cells [2]. The cell protrusion of a leading edge relies on Cdc42 and Rac activities, which are coupled to Rho activity-dependent contractility, supporting the movement of the cell body forward [87]. Consistent with the essential role of the cytoskeleton in promoting cancer migration, its deregulation may cause anti-adhesive and anti-migratory effects. Two snake venom calcium-dependent (C-type) lectins alter the actin/cytoskeleton network in cancer cells. C-type lectins identified from snake venoms are classified in two groups: C-type glycan-binding lectins; and C-type lectin-like proteins, which do not interact with sugars. The C-type glycan-binding lectins are homodimeric non-enzymatic proteins that contain a carbohydrate recognition domain (CRD), binding mainly with galactose [88].

Daboialectin, a low molecular weight C-type lectin isolated from *Daboia russelii* venom, produces morphological changes, including spindle-like shape with loss of cell–cell contacts in lung cancer cells A549 [89]. This snake toxin decreases the mRNA and protein levels of small GTPases Rho and Rac and increases the Cdc42 expression, which is in accordance with remarkable decrease of F-actin content, inhibition of migration and invasion observed in lung cancer cells treated with it [89].

BJcuL is a C-type lectin from *Bothrops jararacussu* venom composed by a disulfide-linked dimer with high affinity for glycoproteins containing β-d-galactosides [90]. BJcuL binds to cancer cells without affecting the adhesion of these cells to fibronectin, laminin, and type I collagen; however, it produces complete actin filament disorganization and disassembly in malignant cells [91]. This toxin does not block the integrin signaling [92], but it binds to cell surface with ECM glycoproteins, such as its substrate d-galactose, promoting the actin disassembles, an event that could accelerate cancer cell detachment from ECM, producing cell death [91].

## 6. Concluding Remarks

Given that malignant cells during metastasis exhibit molecular mechanisms different from those shown by non-metastatic and highly proliferative cancer cells, the conventional cytostatic drugs, which mainly target the cell proliferation, lack effects on the capacity to disseminate and grow in distant sites of metastatic cancer cells. This review highlights the need to search new anti-metastatic drugs. We identified three anti-metastatic mechanisms of action for at least six classes of toxins from snake venoms: (1) inhibition of ECM components-dependent adhesion and migration, (2) inhibition of EMT, and (3) inhibition of migration by alterations in the actin/cytoskeleton network.

These toxins may represent a natural source of molecular scaffolds to design new anti-migratory and anti-invasive agents by obtaining recombinant proteins or small molecules that act as antagonists of integrin signaling or inductors of actin disassembling by binding of cell surface glycoproteins. A selective inhibition of the signaling machinery involved in the cancer cell migration without affect those of migrating non-malignant cells is an important challenge for the new anti-metastatic drugs.

Interestingly, all anti-cancer evaluations on tumorigenic properties—such as proliferation, angiogenesis, invasion, and metastasis of malignant cells—have been performed with toxins isolated from front-fanged snake species, especially from *Viperidae* species; however, the potential therapeutic applications of toxins described from rear-fanged snake species—e.g., [28,30,31,32,93,94,95]—remain unexplored.

An extensive development and conjugation of drug delivery systems with some snake toxins, which has reduced the toxicity and improved the selectivity toward cancer cells [96,97], highlight their promising applications as direct anti-cancer agents or potential tools for the development of novel therapeutic strategies [16]. Finally, the in vivo validation of anti-metastatic effect described on in vitro cancer cell lines is a pending issue for drug discovery from snake toxins.

## Figures and Tables

**Table 1:** Snake toxins that inhibit the adhesion and migration of cancer cells by interaction with ECM components.

Toxin Name	Snake Species	Adhesive Motif	Integrin Target	ECM Ligand	Effect	Ref.
r-Cam-dis recombinant disintegrin	*Crotalus adamanteus*	RGD	αvβ3	laminin-1	Inhibition of adhesion in pancreatic cancer cells	[41]
r-Colombistatins recombinant disintegrin-like domains from Class-III SVMP	*Bothrops colombiensis*	ECD	n.d.	collagen I	Inhibition of adhesion in SK-Mel-28 melanoma cells	[42]
DisBa-01, recombinant disintegrin	*Bothrops alternatus*	RGD	αvβ3	fibronectin	Loss of cell directionality of migrating oral squamous carcinoma cells	[43]
r-mojastn-1, recombinant disintegrin	*Crotalus scutulatus scutulatus*	RGD	αvβ3, α3, and β1,	fibronectin and vitronectin	Inhibition of adhesion and migration of BXPC-3 pancreatic cancer cell line	[44,45]
r-viridistatin-2, recombinant disintigrin	*Crotalus viridis viridis*	RGD	αvβ3	fibronectin and vitronectin	Inhibition of adhesion, migration and invasion of several cancer cell lines	[44,46]
Lebecin, C-type lectin-like protein	*Macrovipera lebetina*	-	αvβ3	fibronectin and fibrinogen	Inhibition of adhesion and migration of MDA-MB-231 breast cancer cells	[47]
PIVL, Kunitz-type serin protease inhibitor	*Macrovipera lebetina transmediterranea*	RGN	αvβ3	fibronectin and fibrinogen	Inhibition of adhesion, migration and invasion of human glioblastoma U87 cells	[48]

n.d.: not determined.
